# Cervicovaginal cytology in patients undergoing pelvic radiotherapy using the Focalpoint system: results from the RODEO study

**DOI:** 10.1186/s13000-014-0231-7

**Published:** 2015-01-16

**Authors:** Maíra Degiovani Stein, José Humberto T G Fregnani, Cristovam Scapulatempo-Neto, Adhemar Longatto-Filho

**Affiliations:** Pathology Department, Barretos Cancer Hospital, Pio XII Foundation, Barretos, Brazil; Molecular Oncology Research Center, Barretos Cancer Hospital, Pio XII Foundation, Barretos, Brazil; Laboratory of Medical Investigation (LIM) 14, Faculty of Medicine, University of São Paulo, 01246-903 São Paulo, Brazil; Life and Health Sciences Research Institute (ICVS), School of Health Sciences, University of Minho, Braga, Portugal; ICVS/3B’s - PT Government Associate Laboratory, Braga Guimarães, Portugal

**Keywords:** Pap test, Radiotherapy, Automation, Focalpoint, SurePath

## Abstract

**Background:**

Evaluate the performance of the Focalpoint system in identifying and classifying cervical cytology alterations from samples collected from patients treated with Radiotherapy (RT).

**Methods:**

The reproducibility of manual and automated screening by cytotechnologists using the BD Focalpoint GS Imaging System was examined. Samples were collected from May 2010 to August 2011.

**Results:**

A total of 378 treated with RT and 8,967 cytology samples from patients without previous RT, were evaluated. The kappa values for cytological diagnoses read manually and automated in cases without previous RT were as follows: < ASC-H vs. ≥ ASC-H = 0.71; < LSIL vs. ≥ LSIL = 0.66; and < HSIL vs. ≥ HSIL = 0.67. The kappa for cytological diagnoses in post-RT women have showed: < ASC-H vs. ≥ ASC-H = 0.71; < LSIL vs. ≥ LSIL = 0.65; < HSIL vs. ≥ HSIL = 0.57.

**Conclusions:**

There was no significant difference among the kappa values we found. Post-RT cytology showed small diagnostic agreement between manual and automated examination.

**Virtual Slides:**

The virtual slide(s) for this article can be found here: http://www.diagnosticpathology.diagnomx.eu/vs/13000_2014_231

## Background

Radiotherapy (RT) is commonly used to treat invasive cervical cancer and other gynaecological malignancies and has high treatment efficacy [1]. RT, however, can induce important changes in cellular morphology that can generate bizarre cell shapes that persist for several years [2]. Despite some controversies, cervicovaginal cytology remains an auxiliary tool for diagnosing gynaecological tumour recurrence after radiotherapy [1]. Nevertheless, most abnormalities related to radiation exposure should be carefully evaluated to avoid misinterpretation [1]. Atypical mitosis, dyskaryosis, severe keratinisation, vacuoles mimicking Chlamydia infection, and koilocytosis-like structures mimicking human papillomavirus (HPV) infection are frequently observed after radiotherapy and represent sources of cytological misdiagnosis [3-5].

The difficulties in interpreting post-RT cervical samples, usually obtained from conventional Pap test smears, were also observed in liquid-based cytology (LBC) preparations. However, patients’ cells are generally well-preserved in LBC samples. Wright and colleagues [5] reported an index of satisfactory samples similar to that of non-irradiated samples (approximately 97%), and associated benign alterations after radiation were found in approximately 50% of cases. Despite the well-known limitations of cytology in precisely categorising cytological findings after RT, the frequency of positive Pap tests for HSIL or squamous cell carcinoma (SCC) after RT is generally low, which minimises the potential errors [6].

Currently, automated screening has been suggested as a useful tool with which to safely accelerate an accurate cytological screening. Although there is limited evidence, the sensitivity of automated screening seems similar to that of manual screening [7]. The available data, however, are conflicting. Nevertheless, to achieve acceptable performance in relation to automated screening, manual screening must be restricted to very low workloads (≤41 slides/day) [8]. The efficacy of the Pap test in revealing cytological abnormalities largely depends on the time spent on the screening in addition to the professional skill of the cytotechnologists [8]. Regardless, automated screening is an important option for an internal quality control system, as the number of cytotechnologists has recently been decreasing, and the HPV vaccine is likely to reduce the accuracy of cytological examination due to the associated decreasing number of cervical abnormalities [9,10]. Consequently, automation seems to be a promising tool to assure the quality of a cytology laboratory and avoid errors. Moreover, positive effects of automation on productivity have been reported [9]. All together, these findings seem realistic for ordinary samples but not necessarily for those from post-RT patients. Preneoplastic changes in women undergoing radiotherapy due to genital cancers were proven to be HPV-related lesions, demonstrating that the candidate HPV assay is a powerful ancillary test for cytology [11].

This study aimed to compare the cytological interpretation of samples collected from women who did or did not undergo previous pelvic RT analysed manually versus samples analysed under Focalpoint (FPGS) guidance.

## Methods

The cytological samples were prepared and examined at the Barretos Cancer Hospital between May 2010 and August 2011. A total of 9,345 cervicovaginal cytologies preserved in SurePath™ solution were analysed. The samples were prepared in the BD PrepStain™ System (Burlington, NC, USA) according to the manufacturer’s instructions.

The manual screening of the slides was routinely performed using light optical microscopes by well-trained cytotechnologists. These slides were re-analysed under guidance of the Focalpoint system (Burlington, NC, USA) and randomly and blindly re-evaluated after 12 months by the same group of cytotechnologists using the automated screening Guided Screening Review Station (GS) of the BD Focalpoint GS Imaging System (FPGS). No additional training was applied for the cytotechnologists between the first and the second round of slides’ review.

Cytology classification followed the Bethesda system [12]. Prevalence ratios were calculated and compared based on their 95% confidence intervals. The kappa coefficients and the respective 95% confidence intervals were calculated to evaluate the diagnostic agreement among the cytotechnologists for both manual and computer-guided screenings.

We also evaluated the frequency and prevalence ratio of cytological results and compare with biopsy CIN 2 +.

All women enrolled in this study signed informed consent forms to participate in the study, which was approved by the Ethics committee of the Barretos Cancer Hospital (id number 244/2009).

## Results

Of the 9,345 liquid-based Pap tests available for this analysis, 378 were collected from women who had been previously treated with RT. Tables 1 and 2 shows the frequency and prevalence ratio of cytological results according to RT status read manually and under FPGS assistance, respectively. Cytological abnormalities were more frequent in the RT arm for both screening tools: manual and automated. In Table 1 the prevalence ratios were significantly higher in the RT group for the following diagnoses: ASC-H + AGC (5.0), LSIL (2.4), and HSIL or worse (3.5) and in Table 2 ASC-H + AGC (4.0), LSIL (1.8), and HSIL or worse (4.8).

**Table 1:** **Number and percentage of cases according to cytology result and previous radiotherapy status read manually**

**Cytology result**	**No previous radiotherapy**	**Previous radiotherapy**	**PR**	**95% CI (PR)**
	**N (%)**	**N (%)**		
Negative	8,589 (95.8)	327 (86.5)	0.9	0.87 – 0.94
ASCUS	72 (0.8)	6 (1.6)	2.0	0.9 – 4.5
ASC-H + AGC	83 (0.9)	17 (4.5)	5.0	2.9 – 8.1
LSIL	107 (1.2)	11 (2.9)	2.4	1.3 – 4.5
HSIL or worse	116 (1.3)	17 (4.5)	3.5	2.1 – 5.7

PR = prevalence ratio (ratio of cytology abnormalities between the groups who did or did not receive previous radiotherapy).

95% CI = 95% confidence interval.

**Table 2:** **Number and percentage of cases according to cytology result and previous radiotherapy status read under FPGS orientation**

**Cytology result**	**No previous radiotherapy**	**Previous radiotherapy**	**PR**	**95% CI (PR)**
	**N (%)**	**N (%)**		
Negative	8,524 (95.1)	316 (83.6)	0.9	0.84 – 0.92
ASCUS	89 (1.0)	13 (3.4)	3.4	1.95 – 6.14
ASC-H + AGC	109 (1.2)	18 (4.8)	4.0	2.40 – 6.38
LSIL	146 (1.6)	11 (2.9)	1.8	0.98 – 3.27
HSIL or worse	99 (1.1)	20 (5.3)	4.8	3.00 – 7.66

Tables 3 and 4 compares the biopsy CIN2+ rates distributed according to RT status and cytology results read manually and under FPGS assistance, respectively. No significant difference was found in the CIN2+ proportion between the groups with and without previous radiotherapy for all cytological categories.

**Table 3:** **Number and percentage of CIN2+ cases based on the biopsies according to cytology result and previous radiotherapy status read manually**

**Cytology result**	**No previous radiotherapy**	**Previous radiotherapy**	**PR**	**95% CI (PR)**
	**N (%)**	**N (%)**		
ASCUS + ASCH + AGC	34 (40.5)	6 (33.3)	0.8	0.4 – 1.7
LSIL	20 (37.0)	1 (25.0)	0.7	0.1 – 3.8
HSIL or worse	77 (74.8)	9 (60.0)	0.8	0.5 – 1.2

PR = prevalence ratio (ratio of cytology abnormalities between the groups who did or did not receive previous radiotherapy).

95% CI = 95% confidence interval.

**Table 4:** **Number and percentage of CIN2+ cases based on the biopsies according to cytology result and previous radiotherapy status read under FPGS orientation**

**Cytology result**	**No previous radiotherapy**	**Previous radiotherapy**	**PR**	**95% CI (PR)**
	**N (%)**	**N (%)**		
ASCUS + ASCH + AGC	41 (32.5)	10 (50.0)	1.54	0.93 – 2.55
LSIL	20 (15.9)	2 (10.0)	0.63	0.16 – 2.49
HSIL or worse	65 (51.6)	8 (40.0)	0.78	0.44 – 1.36

We also evaluated the diagnostic concordance among cytotechnologists in manual and automated screening. Table 5 shows the kappa coefficients according to RT status. There was no difference in the kappa values according to RT status and cytologic abnormality (< ASC-H vs. ≥ ASC-H; < LSIL vs. ≥ LSIL; < HSIL vs. ≥ HSIL).

**Table 5:** **Kappa values between manual and automated screening among cytotechnologists in women who did or did not undergo previous radiotherapy**

	**Diagnoses**					
	**< ASC-H vs. ≥ ASC-H**		**< LSIL vs. ≥ LSIL**		**< HSIL vs. ≥ HSIL**	
	**Kappa**	**95% CI**	**Kappa**	**95% CI**	**Kappa**	**95% CI**
No previous radiotherapy N = 8,967	0.71	(0.67-0.75)	0.66	(0.61-0.71)	0.67	(0.60-0.74)
Previous radiotherapy N = 378	0.71	(0.60-0.82)	0.65	(0.51-0.80)	0.57	(0.38-0.77)

**Figure 1:**
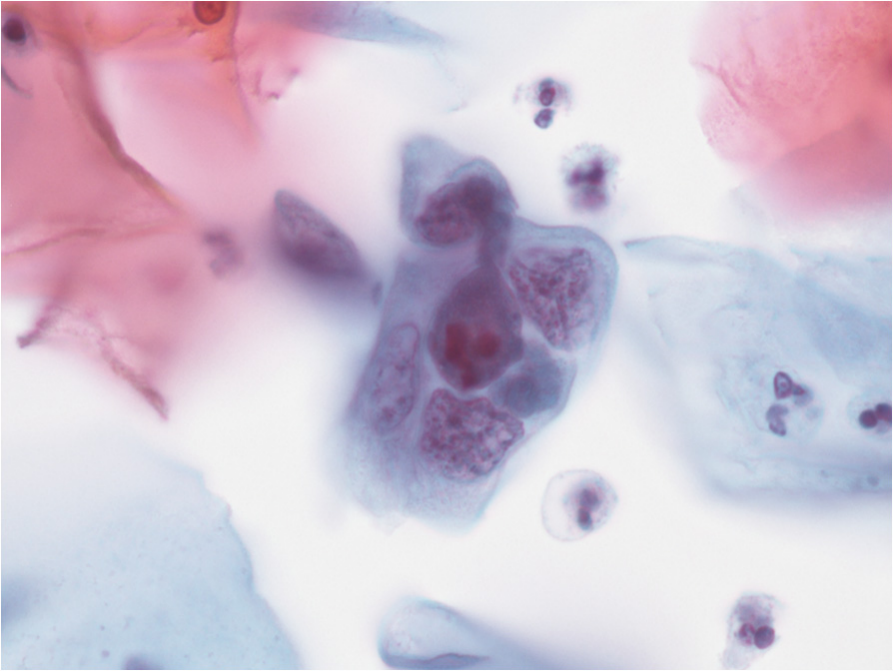
**Multinucleated, bizarre cell with nuclear dyskaryosis resulting from radiation (X100, Papanicolaou stain).**

**Figure 2:**
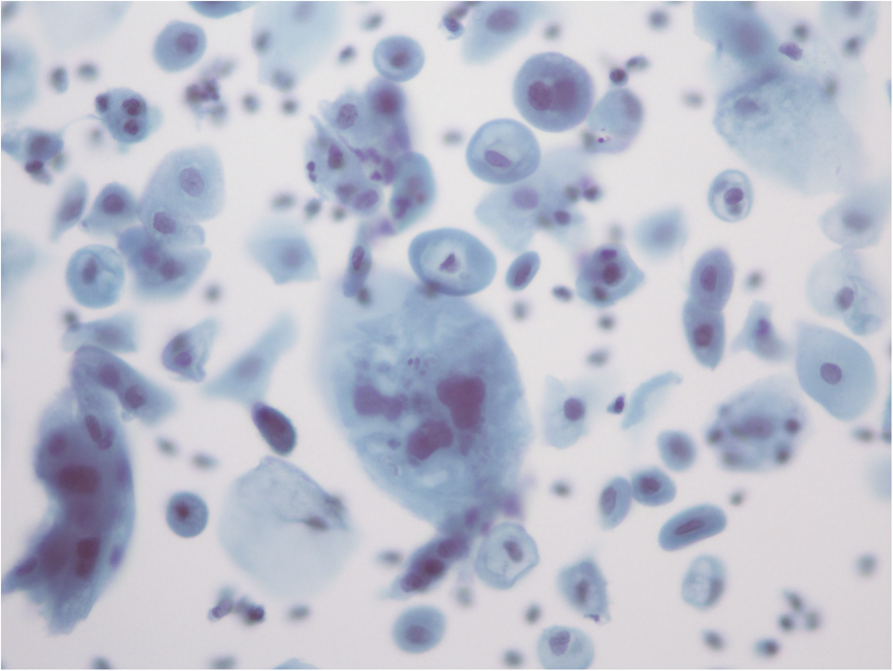
**Post-irradiation isolated cells with an atrophic pattern and slight morphological alterations (X40 magnification, Papanicolaou stain).**

Figures 1 and 2 shows the morphological post-radiotherapy effects on the cells preserved in liquid medium.

## Discussion

The results obtained in this study are unique and original in Brazil. The results demonstrated that previous radiotherapy could introduce important morphological changes that inhibit the specific classification of cellular alterations and favours the cytological categorisation of uncertainty.

In our laboratory, we introduced the concept of internal quality control in cytology supported by an automated method of analysis in order to improve cytological interpretation, theoretically reducing the errors of cytological classification. The subjectivity of manual screening performed by cytotechnologists was examined critically and accurately, and the results obtained in this study provide interesting data to support a significant change in the paradigm of cervical cancer prevention in our hospital, whereby automation can serve concurrently as an option for primary screening and/or as an important tool for internal quality control of cytological diagnosis. Our findings demonstrated, however, that automated screening offers diagnostic performance similar to that of the manual method, even in cases of high-grade lesions, when performed by highly well-trained professionals. These results enabled the automated system FPGS to be introduced into the routine protocol of the cytology laboratory of the Department of Pathology of Barretos Cancer Hospital.

The post-radiation cytology assessment performed in the present study offered substantial data concerning the quality and limitations of cytological examination by automated equipment. The frequencies of lesions found after radiotherapy were as follows: ASC (ASCUS, ASC-H, and AGC), 6.1%; LSIL, 2.9%; and HSIL or worse, 4.5% read manually and read under FPGS orientation were: ASC 8,2%, LSIL 2,9% and HSIL or worse 4,8%. These results are different from those obtained by Wright and colleagues [5], who reported lower frequencies of HSIL or worse than our study (ASCUS, 5.1%; LSIL, 1.0%; HSIL, 0.3%; and carcinoma, 0.7%). Our results are also different from an Italian study, where frequencies ranged from 9.2% for cancer to 13.8% for atypical squamous cells of undetermined significance [13]. These large variations among studies clearly emphasise the difficulties of categorising the effects induced in cervical cells by radiation. The biopsy study of these alterations (Tables 3 and 4), however, reflects the under- and over-estimation that these ionising effects induce, as most of the cytological results are not corroborated by histopathological evaluation. In our study, numerous cases of post-radiation high-grade lesions identified by cytology were not confirmed by biopsy. This finding illustrates that cytological alterations did not correspond to tissue modifications compatible with the diagnosis of intraepithelial lesions. Accordingly, ancillary methods for diagnosis and monitoring of post-radiotherapy patients are necessary to avoid alarming patients and performing unnecessary invasive biopsies.

It is also relevant to emphasise that the manual screening in this study occurred under routine conditions, but for the automated screenings, which were executed almost one year after the initial diagnoses, the cytotechnologists read all the slides blinded of any clinical and laboratorial information of patient (including RT status), which could certainly have influenced the morphological classification. This is especially true for dubious cytological alterations that are frequently found in cases of cell atrophy alterations and post-radiotherapy cytological preparations. For this reason, the analyses of diagnostic reproducibility were performed separately for patients who had and had not undergone RT.

The agreement between the manual and automated screening techniques demonstrated that there was substantial agreement among the cytotechnologists regarding patients who had not undergone RT, but the level of agreement varied significantly for patients who had undergone RT, ranging from substantial agreement with regard to ASC-H and LSIL classifications to moderate agreement for HSIL categorisation. Not surprisingly, the results showed similar kappa values among all observers except for cases of high-grade lesions in patients who had undergone RT, which yielded lower kappa values. Lower concordance from patients’ samples after RT is expected due to the bias that radiation induces, which favours the cytological categorisation of uncertainty.

To the best of our knowledge, no study focusing on FPGS use in post-radiotherapy cervical samples has been reported. Few recent studies evaluating the reproducibility of cytological screening using liquid-based preparations have demonstrated interesting results with image digitalisation. Tsilalis and colleagues [14] studied the performance of a group of pathologists who analysed digital cytology images; this same group reassessed the same images after 12 and 24 months, and the agreement between the readings varied from substantial to excellent, with kappa values varying between 0.79 and 0.97. Through the quality program in Austria, the Pap tests of patients with invasive cervical carcinoma collected in the 5 years prior to disease detection were re-evaluated. The kappa values found between the original and re-assessed readings of the slides were only moderate [15].

A moderate reproducibility has been the most common finding in reports related to the interobserver interpretations of cervical cytological under different scenarios [14]. Our study demonstrated interesting data regarding the performance of cytotechnologists, as most of the results showed substantial reproducibility. Additionally, the significant reproducibility with different kappa values between the two screening techniques shows that the replacement of manual screening with automated screening does not cause loss of positive cases, with high levels of agreement between the manual and automated screening.

Liquid-based cytology has also additional value introducing the molecular biology approach among different lesions from several regions with diverse types of cytological samples. Of note, the relevance of hTERC amplification are feasible to be analysed by FISH technique associated with more progressive CIN3 and carcinoma if present in HPV positive cases in cervical material [16]. Similarly, the genomic amplification pattern of human telomerase RNA gene test was demonstrated to be highly sensitive and suitable for cervical cancer screening in samples preserved in liquid medium, but not C-MYC test because of its lower sensitivity [17]. New options of cellular staining are currently available to improve the quality of the traditional staining protocols observations [18]. In the near future we can speculate that these new staining tools could be also used with the ancillary assistance of computerized screening.

## Conclusion

The potential use of automation in underserved regions is a good option to compensate for the absence of well-trained professionals for slide reading. Moreover, automated reading can improve both the primary screening and/or internal quality control of cytological diagnosis by selecting appropriate fields of observation for cytotechnologists in samples with and without RT effects.
